# Modified eQTL and Somatic DNA Segment Alterations in Esophageal Squamous Cell Carcinoma for Genes Related to Immunity, DNA Repair, and Inflammation

**DOI:** 10.3390/cancers14071629

**Published:** 2022-03-23

**Authors:** Howard H. Yang, Huaitian Liu, Nan Hu, Hua Su, Chaoyu Wang, Carol Giffen, Alisa M. Goldstein, Philip R. Taylor, Maxwell P. Lee

**Affiliations:** 1 Laboratory of Cancer Biology and Genetics, Center for Cancer Research, National Cancer Institute, Bethesda, MD 20892, USA; huaitian.liu@nih.gov; 2 Division of Cancer Epidemiology and Genetics, National Cancer Institute, Bethesda, MD 20892, USA; nhu@nih.gov (N.H.); ssger2002@yahoo.com (H.S.); wangc@mail.nih.gov (C.W.); ag26o@nih.gov (A.M.G.); ptaylor@mail.nih.gov (P.R.T.); 3 Information Management Services, Inc., Silver Spring, Bethesda, MD 20904, USA; giffenc@imsweb.com

**Keywords:** ESCC, eQTL, SNP, DNA segment alterations

## Abstract

**Simple Summary:**

We applied an integrated approach to analyze expression, genotyping and somatic DNA alterations to find genetic markers (genes and SNPs) related to esophageal squamous cell carcinoma (ESCC). We extended the expression-quantitative trait loci (eQTL) analysis by using tumor vs. normal fold change data. By analyzing both RNA and DNA data from multiple platforms and focusing on the genes in three pathways: inflammation, DNA repair, and immunity, we have found results more relevant to ESCC.

**Abstract:**

We integrated ESCC expression and GWAS genotyping, to investigate eQTL and somatic DNA segment alterations, including somatic copy number alteration, allelic imbalance (AI), and loss of heterozygosity (LOH) in ESCC. First, in eQTL analysis, we used a classical approach based on genotype data from GWAS and expression signals in normal tissue samples, and then used a modified approach based on fold change in the tumor vs. normal samples. We focused on the genes in three pathways: inflammation, DNA repair, and immunity. Among the significant (*p* < 0.05) SNP-probe pairs from classical and modified eQTL analyses, 24 genes were shared by the two approaches, including 18 genes that showed the same numbers of SNPs and probes and 6 genes that had the different numbers of SNPs and probes. For these 18 genes, we found 28 SNP–probe pairs were correlated in opposite directions in the two approaches, indicating an intriguing difference between the classical and modified eQTL approaches. Second, we analyzed the somatic DNA segment alterations. Across the 24 genes, abnormal gene expression on mRNA arrays was seen in 19–95% of cases and 26–78% showed somatic DNA segment alterations on Affymetrix GeneChip Human Mapping Arrays. The results suggested that this strategy could identify gene expression and somatic DNA segment alterations for biological markers (genes) by combining classical and modified eQTLs and somatic DNA evaluation on SNP arrays. Thus, this study approach may allow us to understand functionality indicative of potentially relevant biomarkers in ESCC.

## 1. Introduction

Esophageal cancer is the sixth most common cause of cancer death in the world [1]. An estimated 572,034 new esophageal cancer cases and 508,585 deaths occurred in 2018 worldwide, and the new cases and deaths both increased 4% each year between 2012 and 2018 [2,3]. There are two main histologic types of esophageal cancer—esophageal squamous cell carcinoma (ESCC) and esophageal adenocarcinoma. ESCC occurs at particularly high rates in geographic regions that include an East-to-West belt across central Asia and a second belt from eastern to southern Africa. North-Central China (i.e., Shanxi Province) is one of the highest incidence regions for ESCC in the world, and ESCC is the fourth most common cancer in China [4,5]. ESCC has a dismal prognosis, largely because symptoms usually appear late during disease development when the tumors are incurable.

Our previous studies of ESCC conducted in Shanxi Province used a variety of approaches, including population-based epidemiologic and laboratory-based studies of genetic susceptibility and somatic alterations in tumors. Results from these studies suggested that genetic factors, including a positive family history of ESCC, and genomic instability (i.e., high frequency of loss of heterozygosity (LOH)) [6,7,8,9,10,11,12,13], in conjunction with potential environmental exposures played a role in the etiology of ESCC in this high-risk region. Genomic instability is one of several mechanisms leading to gene dysregulation and is thought to play an important role in the etiology of many human cancers.

Genome-wide association studies (GWAS) have emerged as powerful and successful tools to identify common single nucleotide polymorphisms (SNPs) associated with risk of human diseases, including cancers such as ESCC [14,15,16,17]. Although GWAS provided initial insight into genetic variants and susceptibility to cancers, most significant SNPs identified by GWAS are in noncoding regions of genes or in intergenic regions far from genes, making it challenging to determine the functional significance of these SNPs. One approach to investigate the link between GWAS-identified variants and function is to look for variants that influence phenotype, for example, by comparing GWAS variants for differences in gene expression as determined by examination of expression-quantitative trait loci (eQTL). Studies on genome-wide eQTLs in humans can help us to prioritize likely causal variants among the multiple SNPs within the regions identified by GWAS, as well as to reveal the precise biological mechanisms through which DNA sequence differences influence organismal traits [18].

However, there are some challenges for the classical eQTL approach. For example, most SNPs on GWAS are in non-coding regions, and classical eQTL analyses are based on expression signals from normal tissue only and thus only evaluate the relationship between normal expression and genotypes. Thus, classical eQTL does not show relationships between SNPs identified from GWAS and somatic alterations in tumors. In this study, we focused on SNPs/genes that are involved in three pathways—inflammation, DNA repair, and immunity—which are commonly accepted to be related to the etiology of human cancer [19,20,21]. We examined the SNPs/genes in both normal tissue and tumor vs. normal samples to see the relation between biological markers from the classical and modified eQTLs and gene expression in tumor. We also investigated the somatic DNA segment alterations (copy number alterations, allelic imbalance and LOH) on these SNPs/genes in the same group of ESCC patients.

## 2. Materials and Methods

Briefly, cases diagnosed with ESCC between 1994 to 2007 in the Shanxi Cancer Hospital in Taiyuan, Shanxi Province, PR China, and considered candidates for curative surgical resection were identified and recruited to participate in this study after obtaining informed consent. None of the cases had prior therapy, and Shanxi was the ancestral home for all patients.

To better understand the relations among SNPs/genes identified from cancer GWAS and eQTL and somatic DNA segment alterations in tumors, we integrated three types of data: GWAS results from germline DNA [14], genome-wide array results of mRNA expression from tumor and matched normal tissue [22], and genome-wide SNP array results from DNA of tumor and germ line. For this analysis, we focused our evaluation on genes in three pathways: inflammation, DNA repair, and immunity. First, we extracted data from ESCC patients and their matched neighborhood controls from our GWAS study on ESCC [14]. By using the KEGG, Biocarta, and GO databases, we identified 1805 genes in inflammation pathways, 1125 genes in DNA repair pathways, and 735 genes in immunity pathways (Table S6). We selected all loci in these genes, plus 20 Kb around each gene. We also mapped all selected loci on genes present on the Affymetrix Human Genome U133 arrays. Second, we performed eQTL analyses separately by using signals for normal tissue only (called classical) and tumor vs. normal fold change (called modified) in 100 ESCC cases from our previous studies [22] (with GEO accession number GSE44021 for these mRNA array data) for the genes on the three pathways (see Appendix A). Third, we compared significant eQTLs from classical and modified strategies to find loci/genes shared by both approaches. Finally, we examined somatic DNA segment alterations (somatic copy number alterations, LOH, allelic imbalance) in shared genes by classical and modified eQTL analyses in ESCC cases (*n* = 76) by using GeneChip Human Mapping Arrays (GEO accession number for the 500K and SNP 5.0 arrays is GSE74705, and for the SNP 6.0 array, GSE128695). Details of the methods used are described in Appendix A.

## 3. Results

### 3.1. Patient Information

Initially, genome-wide genotyping was performed on a large group of ESCC cases and controls from Shanxi as part of a larger upper gastrointestinal (UGI) cancer GWAS [14]. We have U133 mRNA expression data for 100 ESCC cases, which are a subset of the GWAS samples. Availability of both gene expression and genotype data allowed us to perform eQTL analyses. Characteristics of the 100 ESCC patients evaluated here are summarized in Table S1 as follows: Cases ranged in age from 39 to 71 years (median 58 years) and were predominantly male (60%). Around 26% cases had a positive UGI cancer family history. Clinically, nearly all cases had Stage II tumors (96%) while 46% had evidence of lymph nodes metastasis at diagnosis. Patient survival times ranged from 1.1 months to 67.7 months (median 23.3 months). 

### 3.2. Pathway-Based Analyses

**Figure 1:**
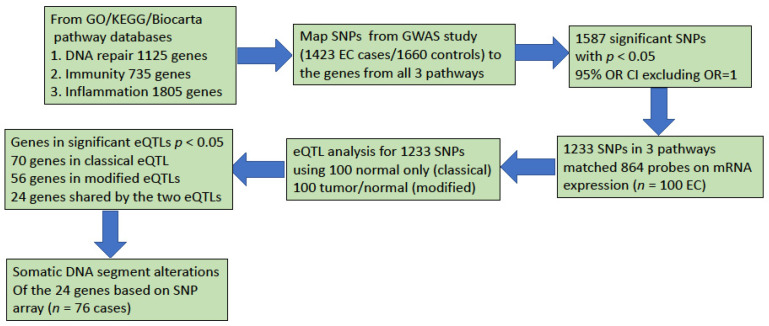
Flow chart for integrated analysis for classical and modified eQTL and somatic DNA segment alterations in immunity, DNA repair and inflammation in ESCC.

From the ESCC GWAS study, we had genotyping data on 550K SNPs for 1423 ESCC cases and 1660 controls. For each SNP, we estimated the odds ratio (OR) and its 95% confidence interval (CI) by using a generalized linear model with adjustment for age and gender. We searched genes in the pathways related to inflammation, immunity, and DNA repair from pathway databases KEGG, Biocarta, and GO. Among the 550K SNPs, 31K were in these genes (or within the 20 Kb 5′ upstream to 20 Kb 3′ downstream window around them). A total of 1587 SNPs showed a nominally significant association with ESCC (*p* < 0.05 and 95% CI did not include OR = 1). Restricting SNPs to genes also on the Affymetrix U133 array reduced the number of significant SNPs from 1587 to 1233 related to 864 genes in the three pathways (Figure 1).

### 3.3. Two eQTL Analyses of Gene Expression and SNP Genotype

#### 3.3.1. Classical eQTL Analysis (in Normal Esophagus Tissue Only)

Although none of the correlations between SNPs and gene expression was significant after Bonferroni correction (0.05/1233 = 4.06 × 10^−5^, there were 131 nominally significant correlations between genotypes of 104 SNPs and expressions of 70 genes with 93 probes in classical eQTL analysis ((a) in Table S2). These eQTLs included 57 positive and 74 negative correlations. Among the positive correlations, the gene–SNP pair CD46 (probe 208783_s_at) and rs7144 had a rho = 0.31 with the smallest *p* = 0.0017; among the negative correlations, the pair CASP8 (probe 213373_s_at) and rs10931936 had a rho = −0.365 and the smallest *p* = 0.0002. Among these eQTLs are several genes with interesting functions, for example ERCC3 and PARD3. DNA repair gene ERCC3 (excision repair cross-complementing), with its product specifically corrected the defect in an early step of the DNA nucleotide excision repair (NER) pathway in UV-sensitive rodent mutants of complementation group 3. ERCC3 (probe 202176_at) was positively correlated with the SNP rs1143407 (rho = 0.26, *p* = 0.009), and the tumor suppressor gene PARD3 (partitioning-defective protein 3), (probe 221280_s_at) was negatively correlated with the SNP rs2496720 (rho = 0.26, *p* = 0.009) (Figure S1a).

There were three patterns among the 131 correlations. With regard to a single gene, we observed correlation(s) between (1) one probe and one SNP; (2) multiple probes and one or two SNPs (i.e., expression of three probes of KLK2 correlated with one SNP); and (3) one probe and multiple SNPs (i.e., CD226, one probe was significantly correlated with five SNPs) ((a) in Table S2).

#### 3.3.2. Modified eQTL Analysis (Fold Change Based on Tumor vs. Normal Tissue)

Modified eQTL analysis identified 114 nominally significant correlations between genotypes of 93 SNPs and expression of 56 genes with 79 probes, including 59 positive and 55 negative correlations ((b) in Table S2). Some of these 56 genes had multiple eQTLs. For example, CDKN2A had two probes (209644_s_at and 207039_at) positively correlated with the SNP rs3731239 (rho = 0.311 and 0.283, respectively), and one probe (211156_at) positively correlated with SNP rs2811708 (rho = 0.230). Furthermore, two probes of BCL2L11 (208536_s_at and 222343_at) were negatively correlated with the SNP rs724710 (rho = 0.274 and −0.246, respectively).

#### 3.3.3. Shared Significant eQTLs in the Classical and Modified Approaches

**Figure 2:**
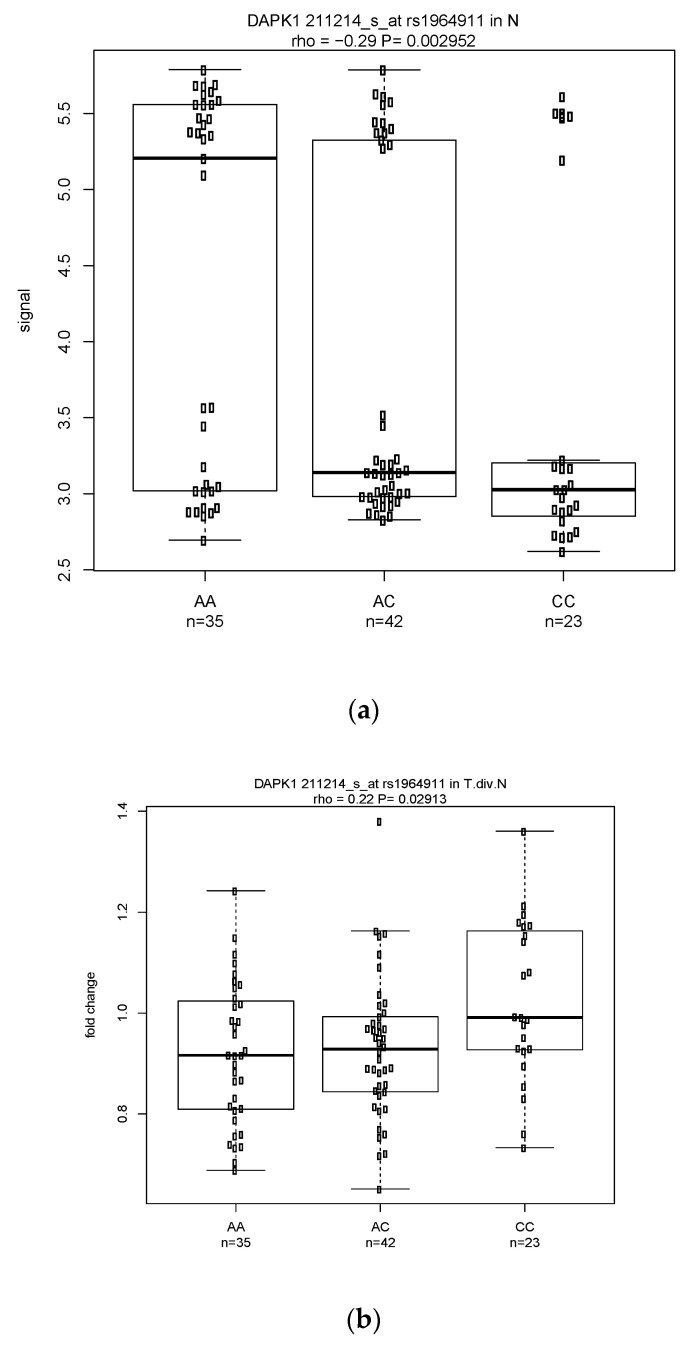
(**a**) Boxplot of the signal in normal and the Spearman correlation between signal and genotype with *p* = 0.003 and a negative rho = −0.29 for the gene–SNP pair *DAPK1* (211214_s_at) and rs1964911. (**b**) Distribution of the tumor vs. normal fold change and the correlation with *p* = 0.03 and a positive rho = 0.22 for the same gene–SNP pair.

**Figure 3:**
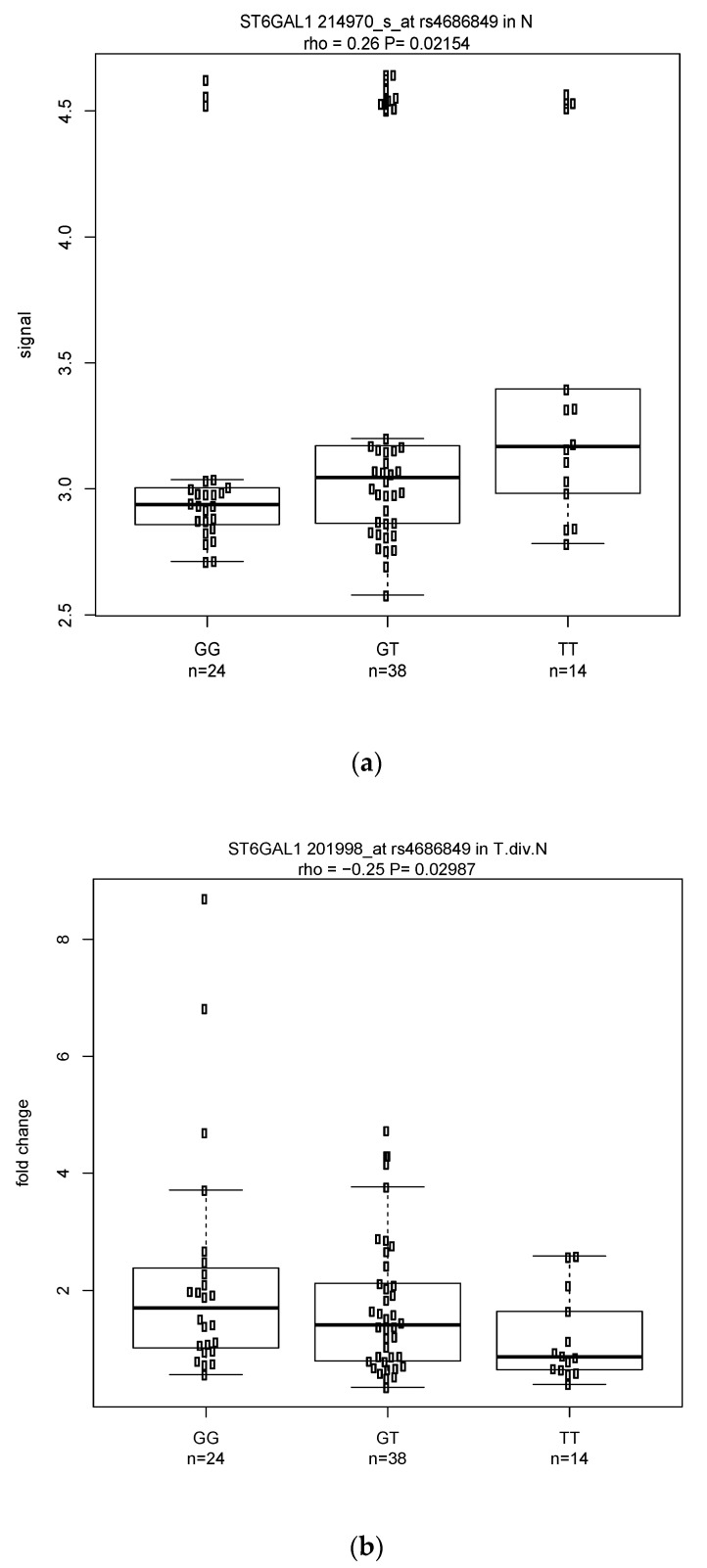
(**a**) Distribution of signal in normal and the correlation with *p* = 0.02 and a positive rho = 0.26 for the gene–SNP pair ST6GAL1 (214970_s_at) rs4686849. (**b**) Distribution of the tumor vs. normal fold change and the correlation with *p* = 0.03 and a negative rho = −0.25 for the same gene–SNP pair.

Among the significant classical (131 eQTLs) and modified (114 eQTLs) eQTLs analyses were 28 eQTL pairs with the same probes and SNPs shared by the two analyses but with effects in opposite directions ((a) in Table 1). These eQTLs included 18 pairs with negative rhos in the classical but positive rhos in the modified approach, and 10 pairs with positive rhos in the classical but negative rhos in the modified eQTL analysis. For example, DAPK1 with probe 211214_s_at and SNP rs1964911 had rho= -0.294 in classical eQTL but rho = 0.218 in modified (Figure 2a,b). Similarly, ST6GAL1 (beta-galactosamide alpha-2,6-sialyltranferase 1) on 3q27 with probe 214971_s_at and SNP rs12495026 also showed opposite directions with rho = −0.242 in classical eQTL (Figure 3a) and 0.22 in modified eQTL (Figure 3b). The same phenomenon was also observed in a gene having one SNP with two probes. For example, SNP (rs2496720) in PARD3 was associated with two probes (221280_s_at and 210094_s_at) whose rho values in classical eQTL (−0.262 and −0.215, respectively) were in the opposite direction as in modified eQTL (0.247 and 0.23, respectively) (Figure S1a,b are plots for 2 of the 4 correlations). There were 18 genes involved in the 28 pairs of eQTLs, including six tumor suppressor genes (DAPK1, PTPRM, GLS2, RARB, TCF7L2, ZBTB16).

### 3.4. Somatic DNA Segment Alterations (Copy Number (CN) Alterations, Allele Imbalance (AI) and LOH) in Genes Shared by the Significant eQTLs from the Two Approaches

We performed advanced investigation of somatic DNA segment alterations at the gene level by using DNAs from tumor and germ line samples. We focused on the genes with significant classical (70 genes, (a) in Table S2) and modified (56 genes, (b) in Table S2) eQTLs. Twenty-four genes were found to be shared by these two groups, including 18 genes already found in the 28 eQTL pairs with the same probes and SNPs ((a) in Table 1) and six genes with different probes and SNPs ((b) in Table 1).

These 24 genes are closely related to ESCC in two aspects. First, 19 of them (Table S5) were differentially expressed genes (DEGs) comparing tumor vs. normal with FDR < 0.05. Second, the survival analysis showed that 7 genes had significant (*p* < 0.05) Kaplan–Meier curves, including 5 genes (MS4A1, N4BP2L1, IGF1R, TCF7L2 and COL11A1) based on the normal expression, and 2 genes (TCF7L1 and PTPRM) based on the tumor expression. Based on the tumor/normal fold change data, the gene IGF1R was found to be significant with a Kaplan–Meier *p*-value of 0.009. The significant Kaplan–Meier plots for three genes—TCF7L1, TCF7L2 and IGF1R, based on normal expression, tumor expression, and tumor to normal fold change—were shown in Figure S2.

Table S3 shows detailed information (pathway involved, and biologic function) on the 24 genes. Fifteen of the 24 genes involved only one of the three pathways under study (inflammation, immunity, and DNA repair), and nine involved two of the three pathways.

**Figure 4:**
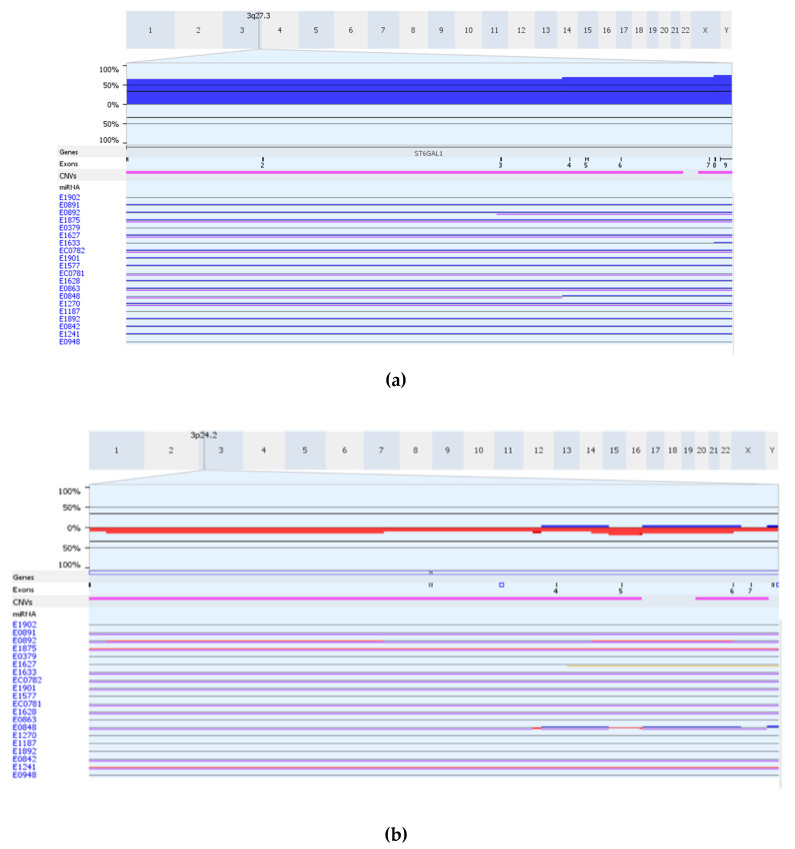
Somatic alteration on ST6GAL1 and RARB in 76 ESCC were analyzed. The copy number change images for two genes, (**a**) ST6GAL1 and (**b**) RARB were shown for 20 ESCC samples as assay examples. On each image, the top portion showed the distribution of somatic alteration and the bottom portion showed individual samples in multiple rows. The colors blue, red, and purple indicated copy number gain, copy number loss, and allelic imbalance, respectively.

Table 2 shows gene expression status and somatic DNA segment alterations of the 24 genes. Among 76 ESCC cases, 19–95% cases had abnormal (over or under) expression across the 24 genes. In addition, 26–78% cases carried DNA segment alterations in the 24 genes. For example, 33% of cases showed overexpression and 68% cases had copy number gains in ST6GAL1. Some genes with mixed CN gain and loss were observed. For example, MS4A1 showed more cases with CN gain than loss. ITPR1 showed more cases with CN loss than gain, and DAPK1 showed CN gain and loss plus mixed LOH. We noted that two genes (CD46, ST6GAL1) showed CN gain only (Figure 4a is an example for CN gain on ST6GAL1), whereas three genes (CYP2C18, CYP2C9, RARB) showed more CN loss (Figure 4b is an example for CN loss on RARB) (Table S4). Furthermore, we noticed that for the 24 genes, the AI ranged from 4% (ST6GAL1 in 3/76) to 43% (PDCD1LG2 in 33/76) of the cases with AI only (Table 2).

We also checked somatic DNA segment alterations in the (32 of 56) genes identified by modified eQTL, which were not shared with classical eQTL, and DNA segment alterations were observed with a range from 26% (ATF6 in 20/76) to 82% (CDKN2A in 62/76) of cases (Table S4). Overall, the results indicated that genes/SNPs selected from classical and modified eQTLs also carried DNA somatic alterations.

## 4. Discussion

By focusing on genes in the three pathways—inflammation, DNA repair, and immunity—and integrating RNA and DNA data, we have obtained results more relevant to ESCC. Classical eQTLs may provide a crucial link between the variants from GWAS research and the biological processes they affect. However, because most of these SNPs are located on non-coding regions of genes, and although the identification of variants that affect phenotypes is rapidly progressing, the current fundamental challenge is to understand how these variants exert their effects. Thus, we applied an approach by combining classical and modified eQTLs to find shared SNP–probes, which were on genes involved in the three pathways (inflammation, DNA repair, immunity). Paired tumor/normal data from the same individuals were used to obtain the modified eQTLs to control for interindividual genetic differences. We also investigated somatic DNA segment alterations for the genes shared by the two kinds of eQTLs from a subset (n = 76) of these ESCC cases by using DNAs from tumor and germline samples using an Affymetrix SNP array. For example, CN variations defined as DNA segments that are 1 kb or larger in size present at variable copy number in comparison with a reference genome and have attracted much attention. It is generally accepted that a somatic CN alteration is highly associated with the development and progression of numerous cancers through its impact on gene expression levels positively or negatively [23,24,25]. Furthermore, AI can arise from the complete loss of an allele or an increase in copy number of one allele relative to the other.

Among the significant classical (131 eQTLs) and modified (114 eQTLs) eQTLs analyses, there were 28 eQTL pairs shared by the two eQTL analyses, but interestingly the 28 pairs of correlations were in opposite directions. The two analyses differed in that the signal of normal mRNA was used in classical eQTL to evaluate associations between SNP–probe(s) in normal tissue whereas the modified eQTL used fold change to connect SNP and probe/gene expressions in tumor. Thus, the opposite direction of the associations observed suggested that the SNP–probe pairs identified in classical eQTL also could potentially influence gene expression in tumor directly/indirectly, suggesting that these SNP–probe pairs may play a role related to gene expression during the development of ESCC. Among the 24 genes ((a), (b) in Table 1) shared by the two eQTL analyses, 79% of them are DEGs and 7 genes had significant (*p* < 0.05) Kaplan–Meier curves. Figure S2 showed the three significant Kaplan–Meier plots for three genes: TCF7L1, TCF7L2, and IGF1R. The patients with higher expression in these three genes had better survival rates. This was consistent with the gene TCF7L2 being one of the tumor suppressor genes. We further examined somatic DNA segment alterations of the 24 shared genes and found that the frequencies of somatic DNA segment alterations, including CN alterations, AI and LOH, in these genes ranged from 26% to 78%. The results indicated that the SNP–probes of the 24 genes in the three pathways may be related to ESCC, although we cannot identify CN gain status for each individual SNP. For example, ST6GAL1 showed somatic CN gain in 68% and gene overexpression in 33% of 76 ESCC examined (Table 2). ST6GAL1 is a member of the family of sialyltransferases, which catalyze the transfer of sialic acids to terminal positions of carbohydrate groups of glycoproteins and glycolipids. ST6GAL1 expression levels have been shown to be upregulated in numerous types of cancers, including pancreatic, prostate, breast, and ovarian cancers, and has been correlated with high tumor grade, metastasis, and reduced patient prognosis in several studies [26]. Aberrant glycosylation is a universal feature of cancer cells, and there is now overwhelming evidence that glycans can modulate pathways intrinsic to tumor cell biology. To date, ST6GAL1 CN gain in ESCC has not been reported in the literature. Thus, the results by using our approach may contribute insight into somatic DNA segment alterations of genes identified based on eQTL studies related to ESCC, in additional to mutations of driver genes.

Our work has several limitations. First, each probe on the mRNA array covered only a small coding region of the gene so that the results do not reflect the full gene expression status (like RNA sequencing). Second, the study had a relatively small sample size resulting in small case numbers in each of the three genotypes. Finally, it was not possible to examine somatic mutations of the 24 genes identified.

In summary, by combining classical and modified eQTL data on genes in three pathways (inflammation, DNA repair, and immunity), we identified 24 genes shared by the two eQTL analyses. The results suggested that by connecting results from classical and modified eQTLs and somatic DNA alterations, it may be possible to better understand functionality indicative of the potentially relevant biomarkers in ESCC by the integrated approach.

## 5. Conclusions

By combining classical and modified eQTL data on genes in three pathways (inflammation, DNA repair, and immunity), we identified 24 genes shared by the two eQTL analyses. We found that majority of the 24 genes are differentially expressed genes comparing tumor to normal and some of them are useful for ESCC patient prognosis prediction. The results suggested that by connecting results from classical and modified eQTLs and somatic DNA alterations, it may be possible to better understand functionality indicative of the potentially relevant biomarkers in ESCC by the integrated approach.

## Figures and Tables

**Table 1:** The two sets of eQTL results from the classical and modified analyses had 24 genes in common. (**a**) Shared 18 of the 24 genes were in 28 probe–SNP pairs with the same probes and SNPs shared by the two sets of results. (**b**) Shared 6 of the 24 genes did not have the same probes and SNPs between the two sets.

(a)										
Gene No	Gene Name	Chr	SNP	Probeset	Classical eQTL		*p* Value	Modified eQTL	*p* Value	SNP
					rho			rho		No
1	CD46	1q32	rs7144	208783_s_at	0.310		0.002	−0.273	0.006	1
			rs2724391	208783_s_at	0.261		0.010	−0.211	0.037	2
2	CD58	1p13	rs1335532	216942_s_at	0.226		0.024	−0.210	0.036	3
3	COL11A1	1p21	rs2061705	37892_at	−0.223		0.028	0.242	0.017	4
4	CYP2C18	10q24	rs1409654	215103_at	−0.321		0.001	0.245	0.014	5
			rs2296679	215103_at	−0.312		0.002	0.240	0.016	6
			rs1409654	208126_s_at	−0.281		0.005	0.221	0.028	7
5	CYP2C9	10q24	rs4086116	214420_s_at	−0.206		0.040	0.209	0.037	8
			rs4917639	214420_s_at	−0.206		0.040	0.209	0.037	9
6	DAPK1	9q21.33	rs1964911	211214_s_at	−0.294		0.003	0.218	0.029	10
7	GLS2	12q13	rs6581096	205531_s_at	−0.201		0.044	0.226	0.024	11
8	N4BP2L1	13q12-q13	rs1207952	211390_at	0.224		0.025	−0.301	0.002	12
9	NCAM1	11q23.1	rs2850303	212843_at	0.271		0.006	−0.355	0.000	13
			rs584427	212843_at	0.251		0.013	−0.314	0.002	14
			rs1821693	212843_at	0.242		0.016	−0.352	0.000	15
10	NCAPD2	12p13.3	rs917634	201774_s_at	−0.337		0.001	0.198	0.048	16
11	PARD3	10p11.22-	rs2496720	221280_s_at	−0.262		0.009	0.247	0.014	17
		p11.21	rs2496720	210094_s_at	−0.215		0.033	0.231	0.022	18
12	PDCD1LG2	9p24.2	rs1360238	220049_s_at	−0.202		0.044	0.295	0.003	19
13	PTPRM	18p11.2	rs12606738	216292_at	−0.202		0.046	0.222	0.028	20
14	TCF7L1	2p11.2	rs12714137	221016_s_at	−0.211		0.037	0.213	0.035	21
15	TCF7L2	10q25.3	rs1028629	212761_at	−0.299		0.003	0.205	0.041	22
16	ZBTB16	11q23.1	rs2852796	205883_at	−0.237		0.017	0.243	0.015	23
17	MS4A1	11q12	rs4939363	210356_x_at	0.216		0.033	−0.224	0.027	24
			rs4939362	210356_x_at	0.199		0.047	−0.202	0.044	25
			rs1941030	210356_x_at	0.199		0.047	−0.202	0.044	26
18	ST6GAL1	3q27-q28	rs12495026	214971_s_at	−0.242		0.016	0.220	0.028	27
			rs12495023	214971_s_at	−0.236		0.018	0.213	0.035	28
**(b)**										
**Gene No**	**Gene**	**Cytoband**	**Classical eQTL** **SNP**	**Probeset**	**rho**	***p*** **Value**	**Modified eQTL** **SNP**	**Probeset**	**rho**	***p*** **Value**
1	CACNA1C	12p13.3	rs2239097	208020_s_at	0.229	0.0222	rs2239097	211592_s_at	−0.208	0.037
			rs2283318	208020_s_at	0.219	0.0297				
2	CACNB2	10p12	rs1034139	215365_at	−0.202	0.0434	rs4748472	207776_s_at	−0.245	0.015
3	IGF1R	15q26.3	rs2684811	203627_at	−0.352	0.0003	rs12908437	208441_at	0.218	0.029
4	ITPR1	3p26-p25	rs11714599	216944_s_at	0.204	0.0431	rs3805032	203710_at	−0.253	0.012
			rs11714599	203710_at	0.204	0.0433	rs3805032	203710_at	−0.253	0.012
							rs304051	216944_s_at	0.250	0.012
							rs304053	216944_s_at	0.250	0.012
							rs304051	222314_x_at	−0.218	0.029
5							rs304053	222314_x_at	−0.218	0.029
	NRP1	10p12	rs4934583	210615_at	−0.213	0.0335	rs869636	210510_s_at	−0.309	0.002
							rs2776928	210510_s_at	−0.231	0.022
							rs2776928	212298_at	−0.215	0.032
6	RARB	3p24	rs922939	208412_s_at	0.227	0.0238	rs12630664	208413_at	−0.293	0.003
			rs17016781	208413_at	−0.220	0.0282	rs12631063	208413_at	−0.271	0.007
							rs3773439	208412_s_at	0.271	0.007
							rs1730223	208412_s_at	0.269	0.007
							rs17016773	208412_s_at	0.266	0.008
							rs11707637	208412_s_at	0.265	0.008
							rs7610831	208412_s_at	0.253	0.011
							rs17029657	208412_s_at	0.239	0.017
							rs6800566	217020_at	0.225	0.024
							rs17016738	208412_s_at	0.216	0.031

**Table 2:** Cross-sample characteristics of gene expression based on mRNA array (*n* = 100) and somatic alterations based on SNP array (*n* = 76) for the 24 genes in ESCC.

No	Gene	Cytoband	Gene Expression: Frequency			Somatic DNA Segment Alterations: Frequency			
			Over	Under	Abnormal	AI Only	CN Gain with/without AI	CN Loss or LOH with/without AI	Total with Alterations
1	CACNA1C	12p13.3	0.06	0.16	0.22	0.13	0.21	0.05	0.39
2	CACNB2	10p12	0.09	0.36	0.45	0.22	0.05	0.16	0.43
3	CD46	1q32	0.14	0.25	0.39	0.24	0.20	0.00	0.43
4	CD58	1p13	0.33	0.13	0.46	0.17	0.03	0.09	0.29
5	COL11A1	1p21	0.95	0	0.95	0.16	0.03	0.12	0.3
6	CYP2C18	10q24	0.07	0.81	0.88	0.24	0.00	0.11	0.34
7	CYP2C9	10q24	0.04	0.62	0.64	0.24	0.00	0.11	0.34
8	DAPK1	9q34.1	0.21	0.22	0.43	0.33	0.12	0.24	0.68
9	GLS2	12q13	0.28	0.18	0.46	0.16	0.08	0.03	0.26
10	IGF1R	15q26.3	0.54	0.03	0.57	0.17	0.18	0.08	0.43
11	ITPR1	3p26-p25	0.09	0.47	0.56	0.24	0.03	0.38	0.64
12	MS4A1	11q12	0.32	0.2	0.52	0.26	0.03	0.05	0.34
13	N4BP2L1	13.q13.1	0.13	0.17	0.3	0.29	0.03	0.28	0.59
14	NCAM1	11q23.1	0.09	0.27	0.36	0.26	0.04	0.18	0.49
15	NCAPD2	12p13.3	0.81	0.05	0.86	0.18	0.14	0.03	0.36
16	NRP1	10p12	0.52	0.08	0.6	0.22	0.07	0.09	0.38
17	PARD3	10p11	0.03	0.47	0.5	0.17	0.07	0.16	0.39
18	PDCD1LG2	9p24.2	0.13	0.08	0.21	0.43	0.05	0.29	0.78
19	PTPRM	18p11.2	0.26	0.09	0.35	0.17	0.16	0.08	0.41
20	RARB	3p24	0.04	0.15	0.19	0.30	0.00	0.36	0.66
21	ST6GAL1	3q27-q28	0.33	0.07	0.4	0.04	0.68	0.01	0.74
22	TCF7L1	2p11.2	0.32	0.27	0.59	0.16	0.13	0.03	0.32
23	TCF7L2	10q25.3	0.17	0.13	0.3	0.24	0.03	0.14	0.41
24	ZBTB16	11q23.1	0.05	0.74	0.79	0.22	0.03	0.21	0.46

## Data Availability

The expression and genotyping data are available at https://www.ncbi.nlm.nih.gov/geo/ (accessed on 20 March 2022) via GEO accession numbers GSE44021 for Affymetrix U133 arrays, and GSE74705 and GSE128695 for SNP arrays.

## Supplementary Materials

The following supporting information can be downloaded at: https://www.mdpi.com/article/10.3390/cancers14071629/s1, Figure S1: (a) Distribution of expression in normal with a negative rho for the gene-SNP pair PARD3 (221280_s_at) and rs2496720; (b) Distribution of tumor vs. normal fold change with a positive rho for the same gene-SNP pair, Figure S2: Significant Kaplan-Meier plots for three genes: TCF7L1, TCF7L2 and IGF1R based on (a) TCF7L2 expression in normal; (b)TCF7L1 expression in tumor; (c) IGF1R expression in normal; (d) IGF1R tumor/normal fold change; Table S1: Clinical characteristics and risk factors in100 ESCC cases, Table S2: (a) eQTL identified significant 70 genes with 93 probes and 104 SNPs in Normal only at *p* < 0.05. (b) eQTL identified significant 56 genes with 79 probes and 93 SNPs in Tumor vs Normal group at *p* < 0.05, Table S3: Information for shared 24 genes by classical and modified eQTLs, Table S4: Somatic DNA segment alterations for 56 genes identified in modified eQTL using SNP array, Table S5: Among the 24 genes in Table 1, we found 19 of them were differentially expressed genes with FDR < 0.05, Table S6: The lists of genes in the inflammation, DNA repair, and immunity pathways.

## Appendix A

### Appendix A.1. Biological Specimen Collection and Processing

#### Appendix A.1.1. U133A Arrays (U133A, U133A v.2.0, and U133 Plus2) and Data Normalization

Gene expression of paired tumor/normal samples was examined by using Affymetrix Human Genome U133 arrays for 100 ESCC cases [22]. Of these 100 paired samples, 37 pairs used the Human U133A chips, 57 pairs used the U133A version 2.0 chips, and 6 pairs used the U133 Plus2 chips from Affymetrix. Probes were prepared according to the protocol provided by the manufacturer (Affymetrix GeneChip expression analysis technical manual), available from: http://www.affymetrix.com/support/index.affx, accessed on 6 March 2022).

For the Affymetrix U133 array data, raw datasets (CEL files on all samples) after scanning were normalized by using Robust Multi-array Average (RMA) [27] implemented in Bioconductor in R (http://www.bioconductor.org, accessed on 6 March 2022), including background correction and normalization across all samples. For each sample, log2 fold changes in gene expression were calculated by subtracting the adjacent normal RMA value from the corresponding tumor RMA value.

#### Appendix A.1.2. GeneChip Human Mapping Arrays (500K, SNP 5.0, and SNP 6.0)

The processing of DNA used in array analyses was described in previous publications [6,13,22]. Tumor and germline DNA from 76 cases (58 of them were in GWAS data, but all had mRNA data) were analyzed by using the Affymetrix 500K Array Set, or SNP 5.0 Array, or SNP 6.0 Array. The detailed gene chip protocols can be found at

http://www.affymetrix.com/support/downloads/manuals/500k_assay_manual.pdf, accessed on 6 March 2022), http://www.affymetrix.com/support/downloads/manuals/genome_wide_snp_5_0_manual.pdf, accessed on 6 March 2022) and http://www.affymetrix.com/catalog/131533/AFFY/GenomeWide+Human+SNP+Array+6.0#1_1, accessed on 6 March 2022).

Experiments were conducted according to protocol l (GeneChip Mapping Assay manual) supplied by Affymetrix, Inc. (Santa Clara, CA, USA).

The set array contained ~262,000 (Nsp I array) and ~238,000 (Sty I array) SNPs (mean probe spacing = 5.8 Kb, mean heterozygosity = 27%) on the 500K and SNP 5.0 Arrays. Approximately 482,200 SNPs on the SNP 6.0 Array were derived from the 500K and SNP 5.0 Arrays.

Genotype calls were generated by GTYPE v. 4.0 software (Affymetrix). Paired germline and tumor DNA from each case were run together in parallel in the same experiment (i.e., same batch, same day). The GEO accession numbers for these data are as follows: 500K and SNP 5.0 Arrays are GSE74705, SNP 6.0 Array is GSE128695.

#### Appendix A.1.3. GeneChip Data Analysis (500K, SNP 5.0, and SNP 6.0 Arrays)

Probe intensity data from Affymetrix SNP arrays were used to identify DNA alterations in the present study. Raw data [.CEL files] for 20 paired samples from the 500K, 36 paired samples from the SNP 5.0, and 20 paired samples from the SNP 6.0 Arrays were loaded by using R/bioconductor and Aroma.affymetrix. The Aroma.affymetrix package was developed to process Affymetrix Copy Number and SNP arrays, and provides estimates of the total copy number for all loci as well as allele B fractions for all (bi-allelic) SNPs. For more details, go to http://www.aroma-project.org/, accessed on 6 March 2022. We used the Copy-number Robust Multichip Analysis (CRMA) function implemented in Aroma to preprocess the data and applied the Circular Binary Segmentation (CBS) method to partition the genome. We combined the segmentation results from all three platforms and imported the results into Nexus Copy Number r 7.5 by using the procedures described in the manual (www.Biodiscovery.com, BioDiscovery, Hawthorne, CA, USA). Nexus was used to generate consistent results across cases in the analysis, to facilitate creation of a single database, and to identify discordances between platforms or software.

Criteria for DNA segment alterations were (1) significance threshold of *p* < 5.0 × 10^−6^, maximum contiguous probe spacing of 1 Mb, and a minimum number of probes per segment of three; (2) copy number (CN) was identified by using log R ratio (LRR) as follows: CN=high gain (LRR ≥ 1.0); CN=gain (0.2 < LRR < 1); CN=high loss (LRR ≤ −1.0); CN=loss (−1 < LRR < −0.2); and (3) allelic imbalance (AI) and loss of heterozygosity (LOH) were identified by using B Allele Frequency (BAF) as follows: AI was called when BAF was between either 0.2 to 0.45 or 0.55 to 0.8; and LOH was identified when BAF was either <0.2 or >0.8.

### Appendix A.2. Pathway Resources

Pathway data were retrieved from three resources or pathway catalogues: BioCarta (http://www.gsea-msigdb.org/gsea/msigdb, accessed on 6 March 2022), the Kyoto Encyclopedia of Genes and Genomes database (KEGG) (http://www.genome.jp/kegg/, accessed on 6 March 2022), and the GO (http://www.geneontology.org, accessed on 6 March 2022) database. These databases are also in the collections C2 and C5 in the Molecular Signatures Database (MSigDB) [11].

The physical location of each autosomal SNP was confirmed as being within a genomic region encompassing 20 Kb 5′ upstream and 20 Kb 3′ downstream of a coding gene for SNPs on the gene expression arrays whose genes were in the pathways related to inflammation, immunity, and DNA repair.

### Appendix A.3. Association and eQTL Analyses

We used GWAS genotyping data for 1423 ESCC cases and 1660 controls [14] to estimate odds ratios (OR) and 95% confidence intervals (CI) for each SNP based on the generalized linear model by using the link function logit with adjustment for gender and age (10-year categories). We initially selected SNPs with *p*-values less than 0.05 and the 95% CIs that did not include 1.00, and then further selected SNPs (n = 27,813) in the 20 K neighborhoods of genes in the pathways related to inflammation, immunity, and DNA repair.

For eQTL analyses, we computed Spearman rank correlations between each SNP and gene pair by using genotyping from blood samples and gene expression from tissue (called “classical” eQTL) and from tumor vs. normal fold change data (called “modified eQTL). SNPs/genes with significant eQTLs were selected based on nominally significant *p*-values (*p* < 0.05). Next, we conducted somatic DNA segment alteration analysis on genes that were shared by significant classical and modified eQTLs.
