# Supplementary material for: Modified eQTL and Somatic DNA Segment Alterations in Esophageal Squamous Cell Carcinoma for Genes Related to Immunity, DNA Repair, and Inflammation
Source: Cancers (Basel). 2022 Mar 23;14(7):1629. doi: 10.3390/cancers14071629 (PMC8996990; doi:10.3390/cancers14071629)
Supplement: Supplementary file 1 [file cancers-14-01629-s001.zip › cancers-1648295-Supplementary.pdf]

Supplementary Materials: Modified eQTL and Somatic DNA Segment Alterations in Esophageal Squamous Cell Carcinoma for Genes Related to Immunity, DNA Repair, and Inflammation

Howard H. Yang, Huaitian Liu, Nan Hu, Hua Su, Chaoyu Wang, Carol Giffen, Alisa M. Goldstein, Philip R. Taylor and Maxwell P. Lee

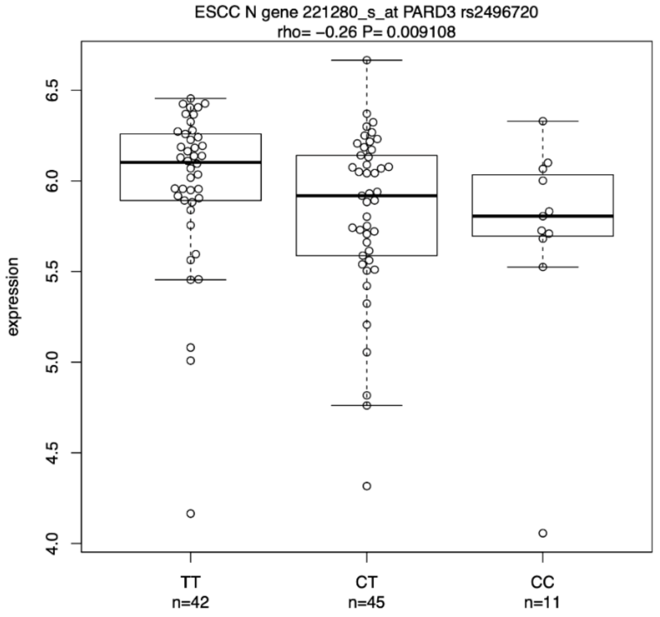

(a)

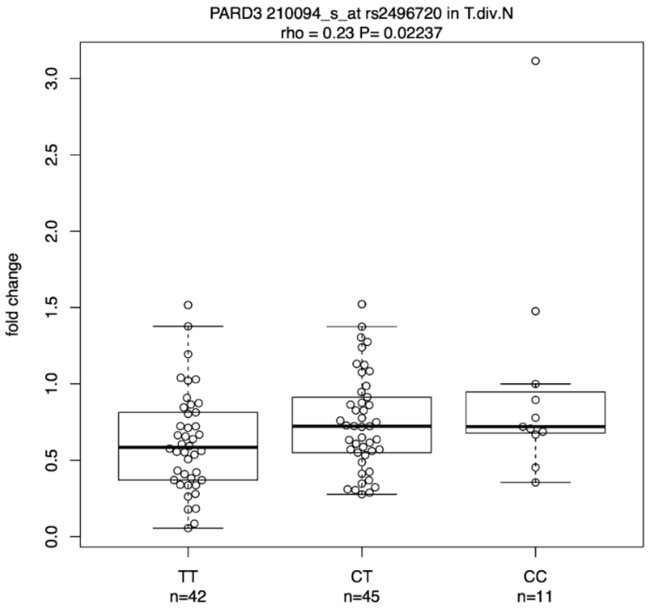

(b)

Figure S1. (**a**) Distribution of expression in normal with a negative rho for the gene-SNP pair *PARD3* (221280_s_at) and rs2496720; (**b**). Distribution of tumor vs. normal fold change with a positive rho for the same gene-SNP pair.

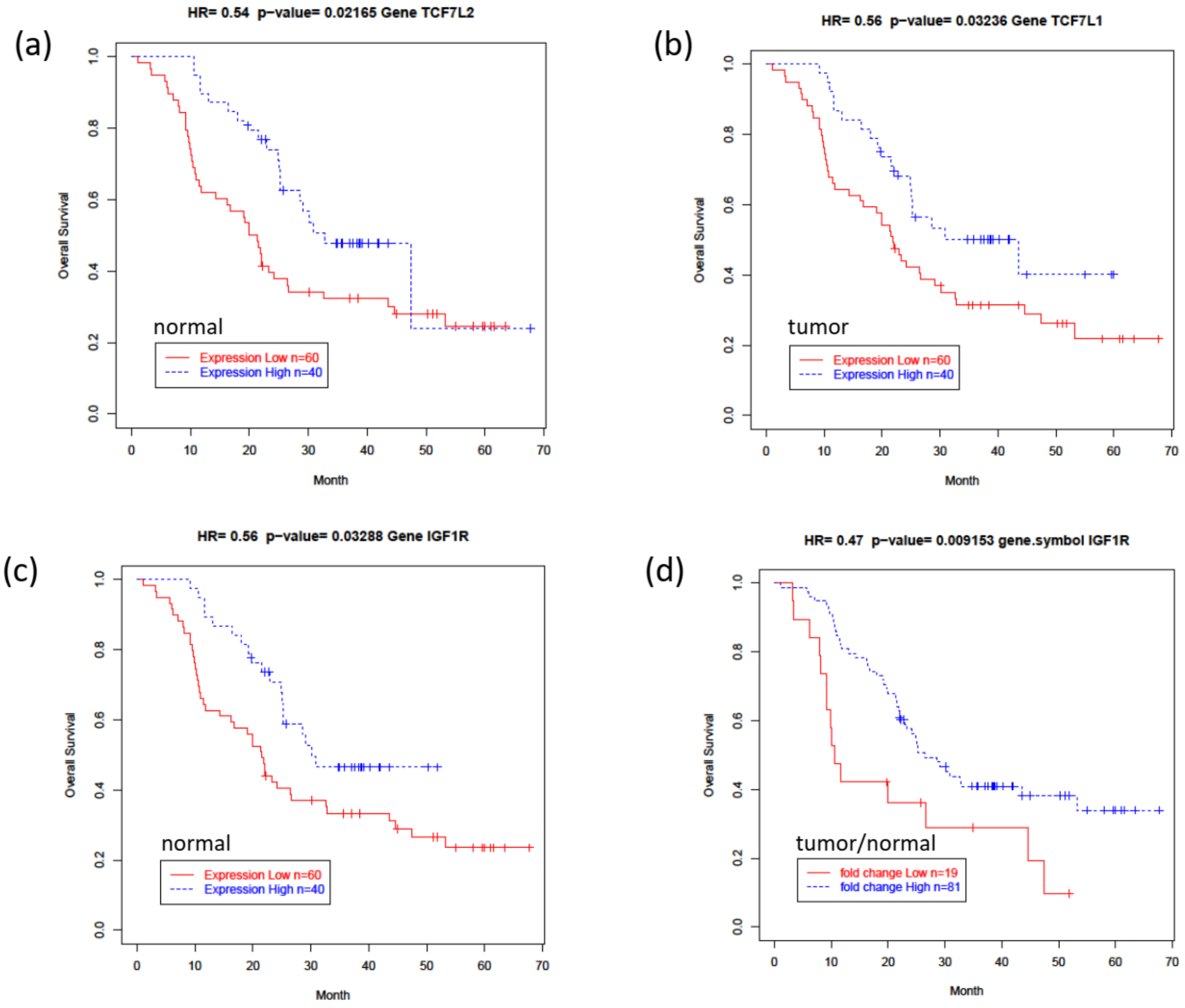

Figure S2. Significant Kaplan-Meier plots for three genes: TCF7L1, TCF7L2 and IGF1R based on (**a**) TCF7L2 expression in normal; (**b**)TCF7L1 expression in tumor; (**c**) IGF1R expression in normal; (**d**) IGF1R tumor/normal fold change.

Table S1. Clinical characteristics and risk factors in100 ESCC cases.

No	Case ID	Age/ Gender	Tumor Location	Stage/ Grade	Metasta-sis (Yes/No)	FH of UGI Cancer (Yse/No)	Smoking (Yes/No)	Alcohol Drinking (Yes/No)	Survival	Survival Months
1	SHE00737	42 / F	Lower	2 / 3	Yes	No	No	No	Alive	42.0
2	SHE00742	61 / M	Middle	2 / 3	Yes	Yes	Yes	No	Deceased	16.5
3	SHE00781	65 / M	Lower	2 / 2	Yes	No	No	No	Alive	43.6
4	SHE00782	49 / F	Middle	2 / 2	Yes	Yes	No	No	Deceased	29.1
5	SHE00796	64 / M	Middle	1 / 3	Yes	No	Yes	Yes	Alive	40.3
6	SHE00816	58 / M	Upper	3 / 3	No	Yes	Yes	No	Deceased	30.2
7	SHE00822	71 / F	Lower	2 / 3	Yes	No	No	No	Deceased	25.0
8	SHE00832	52 / M	Middle	2 / 3	No	Yes	Yes	No	Alive	41.8
9	SHE00845	64 / M	Middle	2 / 3	Yes	No	Yes	No	Deceased	25.3
10	SHE00851	60 / F	Middle	3 / 3	Yes	No	No	No	Deceased	11.6
11	SHE00863	51 / M	Upper	2 / 3	No	No	Yes	Yes	Alive	38.7
12	SHE00864	55 / M	Middle	2 / 4	Yes	No	Yes	Yes	Deceased	10.6
13	SHE00868	68 / F	Middle	2 / 2	No	No	Yes	No	Deceased	28.5
14	SHE00875	48 / F	Middle	2 / 2	No	No	No	No	Alive	39.1

15	SHE00887	56 / M	Middle	1 / 3	No	Yes	No	No	Deceased	24.9
16	SHE00892	67 / F	Middle	3 / 3	No	No	No	No	Alive	37.1
17	SHE00905	58 / F	Middle	2 / 3	No	No	No	No	Alive	34.9
18	SHE00915	62 / M	Middle	2 / 3	Yes	No	Yes	No	Alive	38.8
19	SHE00918	57 / M	Lower	1 / 3	No	No	Yes	Yes	Alive	38.3
20	SHE00920	65 / F	Middle	2 / 3	No	No	No	No	Alive	35.8
21	SHE00921	64 / M	Lower	3 / 3	No	No	Yes	No	Deceased	13.0
22	SHE00925	59 / M	Middle	2 / 3	No	No	No	No	Deceased	25.3
23	SHE00939	67 / F	Middle	3 / 3	No	No	No	No	Alive	34.8
24	SHE00941	40 / M	Middle	3 / 3	Yes	No	Yes	No	Deceased	21.6
25	SHE00942	54 / M	Middle	2 / 3	No	No	Yes	No	Alive	37.6
26	SHE00945	69 / M	Middle	2 / 3	No	No	Yes	No	Alive	37.0
27	SHE00948	62 / F	Middle	3 / 3	Yes	No	No	No	Deceased	26.4
28	SHE00972	49 / F	Lower	2 / 3	Yes	Yes	No	No	Deceased	9.2
29	SHE00973	56 / M	Middle	2 / 2	No	Yes	Yes	No	Unknown	
30	SHE00978	62 / F	Middle	3 / 3	No	No	No	No	Deceased	18.0
31	SHE01204	53 / M	Middle	3 / 2	No	Yes	Yes	Yes	Deceased	30.9
32	SHE01163	69 / M	Middle	2 / 3	Yes	No	Yes	No	Deceased	20.0
33	SHE01179	58 / M	Middle	2 / 3	Yes	Yes	Yes	Yes	Deceased	53.2
34	SHE01187	40 / M	Middle	3 / 3	Yes	No	No	No	Deceased	8.1
35	SHE01195	56 / M	Middle	2 / 3	Yes	No	Yes	No	Alive	30.1
36	SHE01210	52 / M	Middle	2 / 3	No	No	Yes	No	Deceased	43.5
37	SHE01225	51 / M	Lower	2 / 4	Yes	No	Yes	Yes	Deceased	11.6
38	SHE01233	53 / F	Middle	3 / 2	No	Yes	Yes	No	Alive	25.7
39	SHE01242	43 / F	Middle	2 / 2	Yes	No	No	No	Deceased	3.2
40	SHE01255	61 / M	Middle	2 / 2	Yes	Yes	No	Yes	Deceased	19.7
41	SHE01256	65 / F	Middle	2 / 3	No	No	No	No	Alive	35.6
42	SHE01293	60 / M	Lower	2 / 3	Yes	Yes	No	No	Deceased	3.3
43	SHE01301	64 / M	Middle	2 / 3	Yes	Yes	No	No	Alive	22.8
44	SHE01400	59 / M	Middle	3 / 3	Yes	No	Yes	No	Deceased	9.1
45	SHE01405	50 / M	Middle	3 / 3	Yes	No	Yes	No	Alive	22.1
46	SHE01408	63 / M	Middle	2 / 3	No	No	Yes	Yes	Deceased	23.0
47	SHE01415	64 / M	Middle	1 / 3	Yes	No	Yes	Yes	Deceased	44.6
48	SHE01423	53 / F	Middle	2 / 3	Yes	No	No	No	Deceased	10.0
49	SHE01450	39 / M	Middle	3 / 3	Yes	No	Yes	No	Unknown	
50	SHE01451	58 / M	Middle	2 / 3	No	Yes	No	No	Alive	22.2
51	SHE01463	64 / M	Middle	2 / 3	No	No	Yes	No	Deceased	19.3
52	SHE01475	56 / M	Lower	2 / 3	Yes	No	No	No	Deceased	10.5
53	SHE01478	46 / F	Middle	3 / 3	No	Yes	No	No	Deceased	8.0
54	SHE01490	52 / M	Middle	2 / 3	No	No	Yes	No	Alive	19.7
55	SHE01507	62 / F	Middle	2 / 3	Yes	No	No	No	Deceased	21.5
56	SHE01510	63 / F	Middle	2 / 3	Yes	No	No	No	Deceased	23.3
57	SHE01517	64 / M	Middle	2 / 3	Yes	No	Yes	No	Deceased	32.7
58	SHE01520	58 / M	Middle	2 / 2	Yes	No	Yes	No	Alive	63.5
59	SHE01521	53 / F	Middle	2 / 2	No	Yes	No	No	Deceased	16.3
60	SHE01535	50 / F	Middle	2 / 3	No	No	Yes	No	Alive	61.6
61	SHE01542	47 / M	Middle	9 / 3	Yes	No	Yes	Yes	Deceased	21.3
62	SHE01546	57 / M	Middle	2 / 2	No	Yes	Yes	No	Alive	61.0
63	SHE01551	67 / M	Middle	2 / 3	missing	No	Yes	Yes	Alive	58.1
64	SHE01558	52 / F	Middle	3 / 3	No	No	No	No	Deceased	1.1
65	SHE01566	49 / M	Lower	2 / 3	Yes	No	Yes	Yes	Deceased	11.4
66	SHE01572	56 / M	Middle	2 / 2	No	No	No	No	Alive	59.6
67	SHE01573	59 / F	Middle	2 / 3	Yes	No	No	No	Deceased	7.0
68	SHE01576	56 / F	Lower	9 / 2	Yes	No	No	No	Deceased	5.7
69	SHE01577	60 / M	Lower	2 / 3	No	Yes	No	No	Alive	67.7
70	SHE01578	54 / F	Middle	2 / 3	No	No	No	No	Deceased	19.0
71	SHE01584	65 / F	Middle	3 / 3	Yes	No	No	No	Deceased	14.3
72	SHE01589	60 / F	Middle	2 / 3	No	Yes	No	No	Unknown	

73	SHE01610	64 / F	Middle	3 / 3	No	No	No	No	Deceased	10.9
74	SHE01614	45 / F	Middle	2 / 3	No	No	No	No	Alive	51.9
75	SHE01628	54 / M	Middle	2 / 2	missing	No	Yes	No	Deceased	9.9
76	SHE01629	58 / F	Lower	3 / 3	No	Yes	No	No	Deceased	21.8
77	SHE01633	66 / M	Middle	2 / 3	No	No	Yes	No	Deceased	9.7
78	SHE01635	42 / F	Middle	2 / 3	No	Yes	No	No	Alive	50.2
79	SHE01709	49 / F	Middle	2 / 3	No	No	No	No	Deceased	6.0
80	SHE01782	52 / F	Middle	3 / 3	Yes	No	No	No	Deceased	22.1
81	SHE01793	68 / M	Middle	2 / 3	Yes	No	Yes	Yes	Deceased	9.6
82	SHE01796	56 / M	Lower	2 / 3	Yes	Yes	Yes	No	Deceased	10.3
83	SHE01860	50 / M	Middle	2 / 3	No	No	Yes	No	Deceased	47.4
84	SHE01862	49 / M	Middle	2 / 3	No	No	Yes	Yes	Deceased	10.6
85	SHE01864	64 / M	Lower	3 / 3	No	No	No	No	Deceased	9.1
86	SHE01866	58 / M	Middle	3 / 3	Yes	No	Yes	Yes	Alive	51.8
87	SHE01874	53 / F	Middle	3 / 2	Yes	No	No	No	Deceased	6.2
88	SHE01875	64 / M	Middle	2 / 3	No	No	No	Yes	Deceased	22.0
89	SHE01877	62 / M	Middle	2 / 3	Yes	No	Yes	No	Alive	51.2
90	SHE01879	66 / F	Middle	2 / 3	Yes	No	No	No	Deceased	24.1
91	SHE01880	50 / F	Middle	3 / 3	No	No	No	No	Deceased	16.7
92	SHE01882	50 / M	Middle	2 / 2	No	Yes	No	No	Alive	55.1
93	SHE01885	48 / M	Middle	2 / 3	No	No	Yes	No	Deceased	10.8
94	SHE01892	39 / F	Upper	2 / 3	No	No	No	No	Deceased	11.9
95	SHE01897	68 / M	Middle	3 / 3	Yes	No	Yes	No	Deceased	26.6
96	SHE01901	56 / F	Middle	2 / 2	No	No	No	No	Deceased	32.8
97	SHE01902	62 / M	Middle	3 / 3	No	Yes	Yes	No	Alive	60.0
98	SHE01905	65 / M	Middle	2 / 3	No	Yes	Yes	No	Alive	45.0
99	SHE01910	63 / F	Middle	2 / 2	No	No	Yes	No	Alive	38.4
100	SHE02644	40 / M	Middle	2 / 3	No	Yes	Yes	No	Deceased	19.9

Table S2. (**a**) eQTL identified significant 70 genes with 93 probes and 104 SNPs in Normal only at $p < 0.05$. (**b**) eQTL identified significant 56 genes with 79 probes and 93 SNPs in Tumor vs Normal group at $p < 0.05$.

(a)

SNP No	Gene No	Tissuetype	Gene	Cytoband	Gene ID	SNP	Probeset	rho	*p* Value
1	1	N	ADAM10	15q2|15q22	102	rs12908165	214895_s_at	0.204	0.0455
2	2	N	ADCY1	7p13-p12	107	rs2960288	215348_at	−0.223	0.0266
3	3	N	ADCY9	16p13.3	115	rs2239307	204498_s_at	0.217	0.0304
4	4	N	ADIPOR1	1p36.13-q41	51094	rs16850799	217748_at	0.241	0.0164
5	5	N	BCL2L13	22q11	23786	rs1080199	217955_at	−0.228	0.0224
6	6	N	BID	22q11.1	637	rs181396	204493_at	−0.230	0.0214
7		N				rs181396	211725_s_at	−0.214	0.0327
8	7	N	CACNA1C	12p13.3	775	rs2239097	208020_s_at	0.229	0.0222
9		N				rs2283318	208020_s_at	0.219	0.0297
10	8	N	CACNB2	10p12	783	rs1034139	215365_at	−0.202	0.0434
11	9	N	CACNG2	22q13.1	10369	rs2050140	214495_at	0.202	0.0439
12	10	N	CACNG3	16p12-p13.1	10368	rs12598144	206384_at	0.211	0.0360
13	11	N	CASP8	2q33-q34	841	rs10931936	213373_s_at	−0.365	0.0002
14		N	CASP8	2q33-q34		rs3769823	213373_s_at	−0.306	0.0020
15		N	CASP8	2q33-q34		rs10931936	207686_s_at	−0.199	0.0477
16	12	N	CASP9	1p36.3-p36.1	842	rs4646018	203984_s_at	−0.222	0.0263
17		N				rs1052571	203984_s_at	−0.222	0.0263
18		N				rs2042370	203984_s_at	−0.216	0.0321
19		N				rs1862710	203984_s_at	−0.216	0.0321
20	13	N	CCND3	6p21	896	rs12110546	201700_at	0.232	0.0210
21	14	N	CD226	18q22.3	10666	rs1788112	207315_at	−0.274	0.0058
22		N				rs2051322	207315_at	−0.260	0.0091
23		N				rs1788243	207315_at	−0.259	0.0099

24		N				rs10513983	207315_at	−0.243	0.0149
25		N				rs999146	207315_at	−0.229	0.0219
26	15	N	CD28	2q33	940	rs1181390	211856_x_at	−0.299	0.0025
27		N	CD28	2q33		rs3769683	211856_x_at	0.253	0.0114
28	16	N	CD3E	11q23	916	rs2277289	205456_at	−0.257	0.0098
29	17	N	CD46	1q32	4179	rs7144	208783_s_at	0.310	0.0017
30		N				rs7144	207549_x_at	0.273	0.0061
31		N				rs2724391	208783_s_at	0.261	0.0095
32		N				rs7144	211574_s_at	0.248	0.0127
33		N				rs2724391	207549_x_at	0.219	0.0299
34		N				rs2724391	211574_s_at	0.205	0.0433
35	18	N	CD58	1p13	965	rs1335532	216942_s_at	0.226	0.0235
36	19	N	CDH4	20q13.3	1002	rs6089491	220227_at	0.254	0.0112
37		N				rs6089491	206866_at	0.202	0.0453
38	20	N	COL11A1	1p21	1301	rs2061705	37892_at	−0.223	0.0284
39	21	N	COL6A3	2q37	1293	rs3790998	201438_at	0.205	0.0410
40	22	N	COLEC12	18pter-p11.3	81035	rs9954032	221019_s_at	0.249	0.0131
41	23	N	CTBP2	10q26.13	1488 ///	rs2936540	201219_at	0.267	0.0072
42		N		///1p31.3	645291///	rs10901856	201219_at	0.228	0.0227
43		N			650999	rs10794196	201219_at	0.213	0.0345
44		N				rs2946998	201219_at	0.197	0.0497
45	24	N	CYP2C18	10q24	1562	rs1409654	215103_at	−0.321	0.0012
46		N				rs7896133	208126_s_at	−0.314	0.0015
47		N				rs2296679	215103_at	−0.312	0.0016
48		N				rs1409654	208126_s_at	−0.281	0.0049
49		N				rs2296679	208126_s_at	−0.279	0.0050
50	25	N	CYP2C9	10q24	1559	rs4086116	214420_s_at	−0.206	0.0396
51		N				rs4917639	214420_s_at	−0.206	0.0396
52	26	N	DAPK1	9q34.1	1612	rs1964911	211214_s_at	−0.294	0.0030
53	27	N	DDX58	9p12	23586	rs7865082	218943_s_at	0.221	0.0269
54	28	N	EIF2B3	1p34.1	8891	rs10159318	218488_at	0.262	0.0084
55	29	N	ERCC3	2q21	2071	rs1143407	202176_at	0.260	0.0090
56	30	N	FADS1	11q12.2-q13.1	3992	rs174546	208962_s_at	−0.236	0.0187
57		N				rs174556	208962_s_at	−0.234	0.0191
58	31	N	GLS2	12q13	27165	rs6581096	205531_s_at	−0.201	0.0445
59	32	N	HDAC2	6q21	3066	rs9481408	201833_at	0.204	0.0418
60		N				rs3778216	201833_at	−0.203	0.0424
61	33	N	IGF1R	15q26.3	3480	rs2684811	203627_at	−0.352	0.0003
62	34	N	ITGA8	10p13	8516	rs9333185	214265_at	−0.303	0.0021
63	35	N	ITGB1	10p11.2	3688	rs11009157	216190_x_at	−0.242	0.0153
64		N				rs11009157	215879_at	−0.217	0.0304
65	36	N	ITPR1	3p26-p25	3708	rs11714599	216944_s_at	0.204	0.0431
66		N				rs11714599	203710_at	0.204	0.0433
67	37	N	ITPR2	12p11	3709	rs10743591	202660_at	−0.213	0.0330
68	38	N	KLK2	19q13.41	3817	rs198972	210339_s_at	−0.260	0.0089
69		N				rs198972	209854_s_at	−0.260	0.0089
70		N				rs198972	209855_s_at	−0.221	0.0272
71	39	N	LEPR	1p31	3953	rs6673324	207255_at	−0.313	0.0016
72		N				rs4655537	207255_at	−0.285	0.0041
73		N				rs6673324	211354_s_at	−0.260	0.0092
74		N				rs4655537	209894_at	0.222	0.0261
75		N				rs4655537	211354_s_at	−0.221	0.0274
76	40	N	MAP3K4	6q26	4216	rs590988	204089_x_at	0.220	0.0286
77	41	N	MX1	21q22.3	4599	rs469066	202086_at	0.283	0.0043
78		N				rs459498	202086_at	0.265	0.0081
79		N				rs467593	202086_at	0.264	0.0084
80		N				rs2238715	202086_at	0.258	0.0095
81		N				rs457920	202086_at	−0.213	0.0335

82	42	N	N4BP2L1	13q12-q13	90634	rs1207952	211390_at	0.224	0.0250
83	43	N	NCAM1	11q23.1	4684	rs2850303	212843_at	0.271	0.0064
84		N				rs584427	212843_at	0.251	0.0127
85		N				rs1821693	212843_at	0.242	0.0158
86	44	N	NCAPD2	12p13.3	9918	rs917634	201774_s_at	−0.337	0.0006
87		N				rs2072374	201774_s_at	−0.230	0.0220
88	45	N	NRP1	10p12	8829	rs4934583	210615_at	−0.213	0.0335
89	46	N	PARD3	10p11.22-p11.21	56288	rs2496720	221280_s_at	−0.262	0.0091
90		N				rs2496720	210094_s_at	−0.215	0.0331
91	47	N	PDCD1LG2	9p24.2	80380	rs1360238	220049_s_at	−0.202	0.0437
92	48	N	PKP2	12p11	5318	rs7954545	207717_s_at	−0.236	0.0185
93	49	N	PLA2G4A	1q25	5321	rs2076075	210145_at	−0.243	0.0147
94	50	N	PLCB3	11q13	5331	rs2244625	213384_x_at	0.288	0.0036
95	51	N	PPP2CA	5q31.1	5515	rs2292283	215628_x_at	0.202	0.0440
96	52	N	PTPRM	18p11.2	5797	rs12606738	216292_at	−0.202	0.0456
97	53	N	RARB	3p24	5915	rs922939	208412_s_at	0.227	0.0238
98		N				rs17016781	208413_at	−0.220	0.0282
99	54	N	RYR2	1q42.1-q43	6262	rs3753617	207557_s_at	0.222	0.0266
100	55	N	SERPINA1	14q32.1	5265	rs6575424	211428_at	−0.287	0.0040
101		N				rs6575424	211429_s_at	−0.273	0.0062
102		N				rs6575424	202833_s_at	−0.256	0.0104
103	56	N	TCF7L1	2p11.2	83439	rs12714137	221016_s_at	−0.211	0.0367
104		N				rs11126990	221016_s_at	−0.199	0.0469
105	57	N	TCF7L2	10q25.3	6934	rs1028629	212761_at	−0.299	0.0025
106	58	N	TFPI	2q32	7035	rs10153820	213258_at	−0.266	0.0074
107		N				rs10153820	210664_s_at	−0.223	0.0255
108	59	N	TNFAIP3	6q23	7128	rs610604	202643_s_at	0.248	0.0129
109		N				rs610604	202644_s_at	0.223	0.0260
110		N				rs643177	202643_s_at	0.217	0.0298
111		N				rs643177	202644_s_at	0.199	0.0474
112	60	N	TNFRSF1A	chr12p13.2	7132	rs4149623	207643_s_at	0.229	0.0218
113	61	N	TNR	chr1q24	7143	rs1155911	206990_at	0.226	0.0236
114	62	N	TNXB	chr6p21.3	7146 /// 7148	rs3134954	216339_s_at	0.263	0.0082
115		N				rs3134954	211611_s_at	0.219	0.0288
116	63	N	TP73	chr1p36.3	7161	rs3765705	220804_s_at	−0.247	0.0131
117	64	N	VAV2	chr9q34.1	7410	rs2519098	205537_s_at	0.213	0.0334
118	65	N	XPC	chr3p25	7508	rs2733537	209375_at	−0.312	0.0016
119		N				rs1106087	209375_at	−0.238	0.0177
120	66	N	ZBTB16	chr11q23.1	7704	rs2852796	205883_at	−0.237	0.0174
121	67	N	AIF1	chr6p21.3	199	rs2269475	213095_x_at	−0.281	0.0048
122		N				rs2269475	215051_x_at	−0.280	0.0050
123		N				rs2269475	209901_x_at	−0.246	0.0139
124	68	N	MS4A1	chr11q12	931	rs4939363	210356_x_at	0.216	0.0329
125		N				rs4939362	210356_x_at	0.199	0.0470
126		N				rs1941030	210356_x_at	0.199	0.0470
127	69	N	ST6GAL1	3q27-q28	6480	rs12495026	214971_s_at	−0.242	0.0158
128		N				rs12495023	214971_s_at	−0.236	0.0183
129		N				rs12495026	214970_s_at	−0.211	0.0360
130		N				rs12495026	201998_at	−0.199	0.0487
131	70	N	UPF1	19p13.2-p13.11	5976	rs757114	211168_s_at	0.311	0.0017

(b)

SNP No	Gene No	Tissue Type	Gene	Cytoband	Gene ID	SNP	Probeset	rho	*p* Value
1	1	T vs N	ABCC3	17q22	8714	rs4148408	214979_at	0.226	0.024

2	2	T vs N	ADH1B	4q21-q23	124 /// 125 /// 126	rs1229982	209614_at	−0.256	0.010
3	3	T vs N	ALCAM	3q13.1	214	rs3772556	201951_at	−0.242	0.016
4		T vs N				rs3772556	201952_at	−0.241	0.016
5	4	T vs N	ALDH1A3	15q26.3	220	rs4646646	222168_at	−0.205	0.042
6	5	T vs N	ART3	4p15.1-p14	4p15.1-p14	4p15.1-p14	419	rs12504339	210147_at	0.221	0.029
7	6	T vs N	ATF6	1q22-q23	22926	rs905594	217550_at	−0.237	0.018
8	7	T vs N	ATP6V1E1	22pter-q11.2	22q11.1	529	rs5747285	208678_at	−0.206	0.040
9	8	T vs N	BCL2L11	2q13	10018	rs724710	208536_s_at	−0.274	0.006
10		T vs N				rs724710	222343_at	−0.246	0.014
11	9	T vs N	C5	9q33-q34	727	rs7037673	205500_at	0.201	0.045
12	10	T vs N	CACNA1A	19p13.2-p13.1	773	rs2112460	214933_at	0.229	0.023
13		T vs N				rs4926244	206399_x_at	−0.218	0.030
14	11	T vs N	CACNA1C	12p13.3	775	rs2239097	211592_s_at	−0.208	0.037
15	12	T vs N	CACNB2	10p12	783	rs4748472	207776_s_at	−0.245	0.015
16	13	T vs N	CD46	1q32	4179	rs7144	208783_s_at	−0.273	0.006
17		T vs N				rs2724391	208783_s_at	−0.211	0.037
18	14	T vs N	CD58	1p13	965	rs1335532	211744_s_at	−0.216	0.031
19		T vs N				rs1335532	216942_s_at	−0.210	0.036
20	15	T vs N	CDKN2A	9p21	1029	rs3731239	209644_x_at	0.311	0.002
21		T vs N				rs3731239	207039_at	0.283	0.004
22		T vs N				rs2811708	211156_at	0.230	0.022
23	16	T vs N	COL11A1	1p21	1301	rs2061705	37892_at	0.242	0.017
24		T vs N				rs2061705	204320_at	0.215	0.035
25	17	T vs N	CYP2C18	10q24	1562	rs1409654	215103_at	0.245	0.014
26		T vs N				rs2296679	215103_at	0.240	0.016
27		T vs N				rs1409654	208126_s_at	0.221	0.028
28		T vs N				rs2296679	208126_s_at	0.219	0.028
29	18	T vs N	CYP2C9	chr10q24	1559	rs4086116	214420_s_at	0.209	0.037
30		T vs N				rs4917639	214420_s_at	0.209	0.037
31	19	T vs N	DAPK1	9q34.1	1612	rs1964911	211214_s_at	0.218	0.029
32	20	T vs N	DUSP12	1q21-q22	11266	rs3820449	218576_s_at	−0.269	0.007
33	21	T vs N	DYNC2H1	11q21-q22.1	79659	rs7943017	219469_at	0.201	0.045
34	22	T vs N	ELMO1	7p14.2	9844	rs4720230	204513_s_at	−0.258	0.010
35	23	T vs N	FGF12	3q28	2257	rs4687336	207501_s_at	−0.240	0.017
36		T vs N				rs4687336	214589_at	−0.217	0.032
37		T vs N				rs7623367	214589_at	−0.199	0.048
38	24	T vs N	FGF18	5q34	8817	rs6891250	214284_s_at	−0.198	0.048
39	25	T vs N	GLS2	12q13	27165	rs6581096	205531_s_at	0.226	0.024
40	26	T vs N	GNAO1	16q13	2775	rs4783932	204762_s_at	−0.269	0.007
41	27	T vs N	IGF1R	15q26.3	3480	rs12908437	208441_at	0.218	0.029
42	28	T vs N	IL1R1	2q12	3554	rs2041751	202948_at	−0.244	0.015
43	29	T vs N	ITGB5	3q21.2	3693	rs3772839	201124_at	0.201	0.046
44	30	T vs N	ITPR1	3p26-p25	3708	rs3805032	203710_at	−0.253	0.012
45		T vs N				rs304051	216944_s_at	0.250	0.012
46		T vs N				rs304053	216944_s_at	0.250	0.012
47		T vs N				rs304051	222314_x_at	−0.218	0.029
48		T vs N				rs304053	222314_x_at	−0.218	0.029
49	31	T vs N	LAMA2	6q22-q23	3908	rs2571576	205116_at	−0.256	0.010
50		T vs N				rs2571576	216840_s_at	−0.236	0.018
51		T vs N				rs7754167	205116_at	−0.197	0.049
52		T vs N				rs12523864	205116_at	−0.197	0.049
53	32	T vs N	N4BP2L1	13q12-q13	90634	rs1207952	211390_at	−0.301	0.002
54	33	T vs N	NCAM1	11q23.1	4684	rs2850303	212843_at	−0.355	0.000
55		T vs N				rs1821693	212843_at	−0.352	0.000
56		T vs N				rs584427	212843_at	−0.314	0.002
57	34	T vs N	NCAPD2	12p13.3	9918	rs917634	201774_s_at	0.198	0.048

58	35	T vs N	NFATC1	18q23	4772	rs9948543	209664_x_at	0.234	0.021
59	36	T vs N	NLGN1	3q26.31	22871	rs9879266	205893_at	0.234	0.020
60		T vs N				rs976683	205893_at	0.222	0.031
61		T vs N				rs9830510	205893_at	0.212	0.034
62	37	T vs N	NRP1	10p12	8829	rs869636	210510_s_at	−0.309	0.002
63		T vs N				rs2776928	210510_s_at	−0.231	0.022
64		T vs N				rs2776928	212298_at	−0.215	0.032
65	38	T vs N	NTRK2	9q22.1	4915	rs1387924	214680_at	0.289	0.004
66		T vs N				rs1387924	221795_at	0.216	0.031
67	39	T vs N	PARD3	10p11.22-p11.21	56288	rs2496720	221280_s_at	0.247	0.014
68		T vs N				rs2496720	210094_s_at	0.231	0.022
69		T vs N				rs2496720	221526_x_at	0.228	0.024
70	40	T vs N	PDCD1LG2	9p24.2	80380	rs1360238	220049_s_at	0.295	0.003
71		T vs N				rs16923198	220049_s_at	0.240	0.016
72	41	T vs N	PDGFRB	5q31-q32	5159	rs2304060	202273_at	−0.280	0.005
73	42	T vs N	PLCB1	20p12	23236	rs11906514	213222_at	−0.253	0.012
74		T vs N				rs1474683	213222_at	−0.220	0.028
75	43	T vs N	PTPRM	18p11.2	5797	rs12606738	216292_at	0.222	0.028
76	44	T vs N	RARB	3p24	5915	rs12630664	208413_at	−0.293	0.003
77		T vs N				rs12631063	208413_at	−0.271	0.007
78		T vs N				rs3773439	208412_s_at	0.271	0.007
79		T vs N				rs1730223	208412_s_at	0.269	0.007
80		T vs N				rs17016773	208412_s_at	0.266	0.008
81		T vs N				rs11707637	208412_s_at	0.265	0.008
82		T vs N				rs7610831	208412_s_at	0.253	0.011
83		T vs N				rs17029657	208412_s_at	0.239	0.017
84		T vs N				rs6800566	217020_at	0.225	0.024
85		T vs N				rs17016738	208412_s_at	0.216	0.031
86	45	T vs N	SLC7A1	13q12-q14	6541	rs11616506	206566_at	0.361	0.000
87		T vs N				rs10870625	206566_at	0.271	0.007
88		T vs N				rs11616506	212295_s_at	0.254	0.011
89		T vs N				rs2150690	206566_at	0.250	0.012
90		T vs N				rs11616506	212290_at	0.243	0.015
91		T vs N				rs10870625	212295_s_at	0.209	0.038
92		T vs N				rs11616506	215401_at	0.205	0.042
93		T vs N				rs10870625	212290_at	0.203	0.044
94	46	T vs N	STX18	4p16.2	53407	rs2369118	218763_at	−0.263	0.008
95	47	T vs N	TCF7L1	2p11.2	83439	rs6725799	221016_s_at	0.235	0.019
96		T vs N				rs12714137	221016_s_at	0.213	0.035
97	48	T vs N	TCF7L2	10q25.3	6934	rs1028629	212761_at	0.205	0.041
98	49	T vs N	TIAM1	21q22.1|21q22.11	7074	rs2300341	206409_at	0.257	0.010
99	50	T vs N	TNXB	6p21.3	7146 /// 7148	rs3134954	216339_s_at	−0.232	0.020
100		T vs N				rs3134954	208609_s_at	−0.202	0.044
101	51	T vs N	TPM4	19p13.1	7171	rs4808450	212481_s_at	−0.210	0.039
102	52	T vs N	ZBTB16	11q23.1	7704	rs2852796	205883_at	0.243	0.015
103		T vs N				rs589916	205883_at	−0.209	0.037
104		T vs N				rs681200	205883_at	−0.201	0.045
105	53	T vs N	MBP	18q23	4155	rs12456341	210136_at	−0.216	0.032
106		T vs N				rs3794848	210136_at	0.204	0.043
107	54	T vs N	MS4A1	11q12	931	rs4939363	210356_x_at	−0.224	0.027
108		T vs N				rs4939362	210356_x_at	−0.202	0.044
109		T vs N				rs1941030	210356_x_at	−0.202	0.044
110	55	T vs N	OPRK1	8q11.2	4986	rs10504151	207553_at	−0.204	0.043
111		T vs N				rs2303433	207553_at	−0.203	0.046
112	56	T vs N	ST6GAL1	3q27-q28	6480	rs12495023	214971_s_at	0.220	0.028
113		T vs N				rs12495026	214971_s_at	0.213	0.035
114		T vs N				rs2268535	214970_s_at	−0.200	0.046

Table S3. Information for shared 24 genes by classical and modified eQTLs.

No	Gene	Cytobands	Inflamma-tion	Pathway Involved Immunity	DNA Repair	Function Involved
1	CACNA1C	12p13.3	y	y		calcium channel
2	CACNB2	10p12	y	y		calcium channel
3	CD46	1q32	y	y		complement regulatory protein
4	CD58	1p13	y			immunoglobulin superfamily
5	COL11A1	1p21			y	collagen
6	CYP2C18	10q24	y		y	This gene encodes a member of the cytochrome P450 superfamily of enzymes. The cytochrome P450 proteins are monooxygenases which catalyze many reactions involved in drug metabolism and synthesis of cholesterol, steroids and other lipids.
7	CYP2C9	10q24	y		y	cytochrome P450 superfamily of enzymes
8	DAPK1	9q34.1			y	Death-associated protein kinase 1 is a positive mediator of gamma-interferon induced programmed cell death
9	GLS2	12q13	y			a mitochondrial phosphate-activated glutaminase
10	IGF1R	15q26.3	y		y	The insulin-like growth factor I receptor plays a critical role in transformation events.
11	ITPR1	3p26-p25	y			This gene encodes an intracellular receptor for inositol 1,4,5-trisphosphate. Upon stimulation by inositol 1,4,5-trisphosphate, this receptor mediates calcium release from the endoplasmic reticulum. Mutations in this gene cause spinocerebellar ataxia type 15, a disease associated with an heterogeneous group of cerebellar disorders.
12	N4BP2L1	13q12-q13	y			NEDD4 binding protein 2-like 1
13	NCAM1	11q23.1	y			This gene encodes a cell adhesion protein which is a member of the immunoglobulin superfamily. The encoded protein is involved in cell-to-cell interactions as well as cell-matrix interactions during development and differentiation. The encoded protein has been shown to be involved in development of the nervous system, and for cells involved in the expansion of T cells and dendritic cells which play an important role in immune surveillance.
14	NCAPD2	12p13.3	y			non-SMC condensin I complex subunit D2
15	NRP1	10p12	y		y	This gene encodes one of two neuropilins, which contain specific protein domains which allow them to participate in several different types of signaling pathways that control cell migration.
16	PARD3	10p11.22-p11.21	y			This gene encodes a member of the PARD protein family. PARD family members interact with other PARD family members and other proteins; they affect asymmetrical cell division and direct polarized cell growth.
17	PDCD1LG2	9p24.2	y			programmed cell death 1 ligand 2
18	PTPRM	18p11.2	y			The protein encoded by this gene is a member of the protein tyrosine phosphatase (PTP) family. PTPs are known to be signaling molecules that regulate a variety of cellular processes including cell growth, differentiation, mitotic cycle, and oncogenic transformation.
19	RARB	3p24			y	This gene encodes retinoic acid receptor beta, a member of the thyroid-steroid hormone receptor superfamily of nuclear transcriptional regulators. This receptor localizes to the cytoplasm and to subnuclear compartments. It binds retinoic acid, the biologically active form of vitamin A which mediates cellular signalling in embryonic morphogenesis, cell growth and differentiation. It is thought that this protein limits growth of many cell types by regulating gene expression.
20	TCF7L1	2p11.2	y		y	This gene encodes a member of the T cell factor/lymphoid enhancer factor family of transcription factors. These transcription factors are activated by beta catenin, mediate the Wnt signaling pathway and are antagonized by the transforming growth factor beta signaling pathway. The encoded protein contains a high mobility group-box

						DNA binding domain and participates in the regulation of cell cycle genes and cellular senescence.
21	TCF7L2	10q25.3	y		y	This gene encodes a high mobility group (HMG) box-containing transcription factor that plays a key role in the Wnt signaling pathway.
22	ZBTB16	11q23.1			y	This gene is a member of the Krueppel C2H2-type zinc-finger protein family and encodes a zinc finger transcription factor that contains nine Kruppel-type zinc finger domains at the carboxyl terminus. This protein is located in the nucleus, is involved in cell cycle progression, and interacts with a histone deacetylase.
23	MS4A1	11q12		y		This gene encodes a member of the membrane-spanning 4A gene family. Members of this nascent protein family are characterized by common structural features and similar intron/exon splice boundaries and display unique expression patterns among hematopoietic cells and nonlymphoid tissues. This gene encodes a B-lymphocyte surface molecule which plays a role in the development and differentiation of B-cells into plasma cells.
24	ST6GAL1	3q27-q28		y		This gene encodes a member of glycosyltransferase family 29. The encoded protein is a type II membrane protein that catalyzes the transfer of sialic acid from CMP-sialic acid to galactose-containing substrates. The protein, which is normally found in the Golgi but can be proteolytically processed to a soluble form, is involved in the generation of the cell-surface carbohydrate determinants and differentiation antigens HB-6, CD75, and CD76.

Table S4. Somatic DNA segment alterations for 56 genes identified in modified eQTL using SNP array.

No	Gene (Physical Location)	Case# with AI Only	Case# with AI & CN Gain	Case# with AI & CN Loss	Case# with CN Gain	Case# with CN Loss	Case# with LOH	Case# LOH with CN Gain or Loss	Total Case# with Alterations
	shared 24 genes								
1	CACNA1C(chr12:2,032,676-2,677,376)	10	8	4	8	0	0		30
2	CACNB2(chr10:18,469,611-18,870,694)	17	4	8	0	3	1		33
3	CD46(chr1:205,992,005-206,035,484)	18	5	0	10	0	0		33
4	CD58(chr1:116,858,678-116,915,238)	13	1	5	1	2	0		22
5	COL11A1(chr1:103,114,610-103,346,640)	12	1	6	1	0	2	1 mixed (CN gain, loss)	23
6	CYP2C18(chr10:96,433,240-96,485,937)	18	0	5	0	1	2		26
7	CYP2C9(chr10:96,688,404-96,739,138)	18	0	5	0	1	2		26
8	DAPK1(chr9:89,301,962-89,513,369)	25	7	13	2	1	3	1 mixed(gain &LOH)	52
9	GLS2(chr12:55,150,994-55,168,465)	12	2	2	4	0	0		20
10	IGF1R(chr15:97,009,290-97,325,282)	13	7	3	7	1	2		33
11	ITPR1(chr3:4,510,031-4,864,524)	18	0	23	2	4	2		49

12	MS4A1(chr11:59,979,857-59,994,801)	20	0	1	2	1	2		26
13	N4BP2L1(chr13:31,872,859-31,900,315)	22	1	15	1	4	2		45
14	NCAM1(chr11:112,337,178-112,654,368)	20	2	9	1	4	1		37
15	NCAPD2(chr12:6,473,558-6,511,393)	14	5	2	6	0	0		27
16	NRP1(chr10:33,506,424-33,664,012)	17	4	5	1	1	1		29
17	PARD3(chr10:34,438,493-35,144,259)	13	5	8	0	1	2	1 mixed (CN gain & loss)	30
18	PDCD1LG2(chr9:5,500,544-5,561,282)	33	3	15	1	3	3	1 mixed (gain&LOH)	59
19	PTPRM(chr18:7,557,313-8,396,859)	13	8	2	4	2	1	1 mixed (high CN gain&LOH)	31
20	RARB(chr3:25,190,826-25,614,426)	23	0	21	0	1	4	1 mixed (high CN,gain, loss & homozygous loss)	50
21	ST6GAL1(chr3:188,131,008-188,279,035)	3	28	0	24	0	0	1 mixed (CN gain &LOH)	56
22	TCF7L1(chr2:85,214,093-85,391,022)	12	8	1	2	0	0	1 mixed (CN gain, AI &LOH)	24
23	TCF7L2(chr10:114,699,998-114,917,426)	18	1	7	1	2	2		31
24	ZBTB16(chr11:113,435,640-113,626,607)	17	1	11	1	4	1		35
	NOT shared 32 genes								
25	ABCC3(chr17:46,067,216-46,124,062)	36	4	2	2	0	2	0	46
26	ADH1B(chr4:100,446,549-100,461,622)	26	1	8	1	2	1	1 (LOH with loss)	40
27	ALCAM(chr3:106,568,246-106,778,447)	7	16	1	23	0	0	1 (LOH with gian)	48
28	ALDH1A3(chr15:99,237,419-99,274,353)	16	8	2	6	0	2	0	34
29	ART3(chr4:77,151,356-77,252,979)	26	1	11	1	2	1	1(with loss)	43
30	ATF6(chr1:160,002,657-160,200,484)	19	9	1	8	0	0	0	37
31	ATP6V1E1(chr22:16,454,902-16,491,588)	11	3	8	6	2	0	0	30
32	BCL2L11(chr2:111,594,961-111,642,493)	15	2	1	1	1	0	0	20
33	C5(chr9:122,754,434-122,852,375)	32	7	7	2	0	4	1(with gain)	53
34	CACNA1A(chr19:13,178,256-13,478,274)	17	6	5	4	2	1	0	35
35	CDKN2A(chr9:21,957,750-21,984,490)	19	0	10	1	28	1	3 (2 with loss and 1 with gain))	62
36	DUSP12(chr1:159,986,204-159,993,576)	19	6	1	7	0	0	0	33
37	DYNC2H1(chr11:102,485,369-102,855,801)	10	8	8	4	2	1	5 (2 with high CN gain, 2 with high gain and 1 with loss	38
38	ELMO1(chr7:36,859,035-37,455,454)	12	9	1	11	1	0	0	34
39	FGF12(chr3:193,339,875-193,928,082)	6	28	0	25	0	0	0	60

40	FGF18(chr5:170,779,271-170,817,235)	15	0	7	1	0	2	1 (with loss)	26
41	GNAO1(chr16:54,782,751-54,948,857)	17	4	3	4	0	0	2 (loss &LOH)	30
42	IL1R1(chr2:102,053,267-102,162,766)	16	5	1	2	1	1	0	26
43	ITGB5(chr3:125,964,484-126,088,834)	5	22	0	23	0	0	1 (LOH & gain)	51
44	LAMA2(chr6:129,245,978-129,879,403)	16	6	1	4	0	1	0	28
45	MBP(chr18:72,819,776-72,973,762)	19	0	11	1	2	0	0	33
46	NFATC1(chr18:75,256,759-75,390,311)	21	0	12	3	3	0	0	39
47	NLGN1(chr3:174,598,931-175,483,833)	3	31	0	22	0	0	2 (LOH & CN gain,)	58
48	NTRK2(chr9:86,473,192-86,831,805)	34	7	6	3	0	2	2 (gian & LOH)	54
49	OPRK1(chr8:54,300,828-54,326,810)	10	22	0	13	0	0	1(loss, AI &LOH)	46
50	PDGFRB(chr5:149,473,594-149,515,615)	15	1	7	3	0	3	1 (loss &LOH)	30
51	PLCB1(chr20:8,060,911-8,813,547)	11	15	1	9	1	1	0	38
52	SLC7A1(chr13:28,981,550-29,067,825)	25	2	15	1	3	2	2 (loss &LOH)	48
53	STX18(chr4:4,471,596-4,594,676)	26	0	16	0	2	1	1(loss, AI &LOH)	46
54	TIAM1(chr21:31,412,606-31,854,161)	16	0	6	7	4	0	0	33
55	TNXB* (chr6:32,116,910-32,185,129)	19	5	1	4	1	0	0	30
56	TPM4(chr19:16,039,316-16,074,813)	17	1	1	0	3	0	0	22

*There were 25 genes shared between 70 genes in Normal only and 56 genes in tumor vs normal. However, one gene TNXB is a pseudogene and the structure of this gene is unusual in that it overlaps the CREBL1 and CYP21A2 genes at its 5' and 3' ends, respectively. Thus, we dropped TNXB from comparsion analysis for alterations of gene expression and somatic DNA.

Table S5. Among the 24 genes in Table 1, we found 19 of them were differentially expressed genes with FDR < 0.05.

Gene_Symbol	chr	Cytoband	*p* Value	Fold.Change	*q* Value
COL11A1	1	1p21	3.20×10^{-26}	6.749	2.91×10^{-25}
NCAPD2	12	12p13.3	5.62×10^{-24}	1.928	3.14×10^{-23}
CYP2C18	10	10q24	2.98×10^{-21}	0.2084	9.46×10^{-21}
ZBTB16	11	11q23.1	4.49×10^{-15}	0.4226	5.42×10^{-15}
PARD3	10	10p11.22-p11.21	4.70×10^{-13}	0.6115	4.41×10^{-13}
CYP2C9	10	10q24	3.03×10^{-11}	0.7503	2.21×10^{-11}
ITPR1	3	3p26-p25	1.67×10^{-7}	0.6731	7.77×10^{-8}
NRP1	10	10p12	6.14×10^{-7}	1.384	2.64×10^{-7}
IGF1R	15	15q26.3	2.05×10^{-6}	1.349	7.97×10^{-7}
CD46	1	1q32	8.03×10^{-6}	0.7553	3.02×10^{-6}
CACNB2	10	10p12	0.000294	0.6953	8.71×10^{-5}
ST6GAL1	3	3q27-q28	0.001028	1.271	0.000292
NCAM1	11	11q23.1	0.006434	0.7973	0.001593
CD58	1	1p13	0.01376	1.145	0.003295
TCF7L2	10	10q25.3	0.01406	1.112	0.00336

PTPRM	18	18p11.2	0.01672	1.165	0.003949
RARB	3	3p24	0.05956	0.9075	0.01131
CACNA1C	12	12p13.3	0.07267	0.9279	0.01441
DAPK1	9	9q34.1	0.08618	0.9157	0.01679
